# A Staging Scheme for the Development of the Moth Midge *Clogmia albipunctata*

**DOI:** 10.1371/journal.pone.0084422

**Published:** 2014-01-07

**Authors:** Eva Jiménez-Guri, Karl R. Wotton, Brenda Gavilán, Johannes Jaeger

**Affiliations:** EMBL/CRG Research Unit in Systems Biology, Centre for Genomic Regulation (CRG), Universitat Pompeu Fabra (UPF), Barcelona, Spain; University of Otago, New Zealand

## Abstract

Model organisms, such as *Drosophila melanogaster*, allow us to address a wide range of biological questions with experimental rigour. However, studies in model species need to be complemented by comparative studies if we are to fully understand the functional properties and evolutionary history of developmental processes. The establishment of new model organisms is crucial for this purpose. One of the first essential steps to establish a species as an experimental model is to carefully describe its life cycle and development. The resulting staging scheme serves as a framework for molecular studies, and allows us to homologise developmental processes between species. In this paper, we have characterised the life cycle and development of an emerging non-drosophilid dipteran model system: the moth midge *Clogmia albipunctata*. In particular, we focus on early embryogenesis (cleavage and blastoderm cycles before gastrulation), on formation and retraction of extraembryonic tissues, and on formation of the germ line. Considering the large evolutionary distance between the two species (approximately 250 million years), we find that the development of *C. albipunctata* is remarkably conserved compared to *D. melanogaster*. On the other hand, we detect significant differences in morphology and timing affecting the development of extraembryonic tissues and the germ line. Moreover, *C. albipunctata* shows several heterochronic shifts, and lacks head involution and associated processes during late stages of development.

## Introduction

Comparative studies are essential to understand the function and evolution of developmental processes (see the accompanying paper by Wotton *et al.* [1]). To enable such studies, it is necessary to establish new model organisms, which can be used for the experimental investigation of embryogenesis [2]. An important prerequisite for establishing an experimental model is to obtain a detailed and systematic staging scheme describing the timing, dynamics, and spatial arrangement of morphogenetic processes during development. Such a staging scheme forms a frame of reference into which molecular experimental findings can be placed. In this and the accompanying paper, we provide a detailed developmental schedule, staging scheme, and morphological characterisation of the life cycle of two non-drosophilid dipteran species that we use as experimental models in our laboratory: the scuttle fly *Megaselia abdita* [1], and the moth midge *Clogmia albipunctata* (this paper).

The moth midge *C. albipunctata*—also commonly known as the drain fly—is one of the most familiar representatives of the family Psychodidae. Although there has been recent controversy over the phylogenetic relationships of Psychodidae [3], they are likely the sister group of the Neodiptera, a monophyletic taxon formed by the Brachycera—‘higher flies’ including the Drosophilidae and Phoridae—and Bibionomorpha [4], [5], from which they diverged approximately 250 million years ago [3], [5]. Over 3,000 species of moth midges have been described and grouped into six subfamilies [6]. Most species are cosmopolitan, nocturnal inhabitants of aquatic or semiaquatic habitats, although some groups, like sand flies, are able to live in less moist environments. Most moth midge adults have reduced mouthparts but some species of sand flies and sycoracines feed on vertebrate blood. Some of these blood-feeding species can transmit diseases such as leishmaniasis and are dangerous to humans. Other species can become pests when they reach high densities in places like mushroom farms, although sheer numbers can also be beneficial when moth midges grow feeding on biofilms, for example on the filters of sewage treatment plants, preventing them from clogging [6].

Over the past few years, *C. albipunctata* has been the subject of a number of developmental studies. While one early investigation [7] focused on head segmentation, most other papers on the development of *C. albipunctata* target segment determination in the trunk region of the embryo. Rohr *et al.* [8] found that in *C. albipunctata* anterior expression of the gap gene *hunchback (hb)* is conserved compared to the vinegar fly *Drosophila melanogaster*, but posterior expression domains, such as the posterior *hb* domain, the 7th stripe of the pair-rule gene *even-skipped (eve)*, and the posterior stripes of the segment-polarity gene *engrailed (en)*, are delayed until after gastrulation. Stauber *et al.* [9] reported the presence of maternal expression of the Hox3-ortholog *zerknüllt (zen)* but could not find any expression of the maternal factor *bicoid (bcd)*, a gene we now know is missing from the *C. albipunctata* genome (our unpublished data). Bullock *et al*. [10] studied altered pair-rule mRNA localisation in *C. albipunctata* (with a thin blastoderm layer) compared to *D. melanogaster* (where the blastoderm is thicker). Garcia-Solache et al. [11] provided an initial morphological description of a number of developmental landmarks, and a systematic qualitative description of gap gene expression patterns in *C. albipunctata.* Jiménez-Guri *et al.* [4] analysed early embryonic transcriptomes from *C. albipunctata* and other species. Finally, Wotton *et al.* [12] studied the evolution and expression patterns of BMP signalling components. However, none of these studies provide a detailed and systematic characterisation and analysis of the development of *C. albipunctata*, and there is no staging scheme comparable to the one established earlier for *D. melanogaster* (reviewed in [13]).

In this paper, we present an overview of the *C. albipunctata* life cycle, as well as a description of its embryonic development. This effort goes beyond an earlier study of *C. albipunctata* development [11] in its level of detail and the proposal of a standardised staging scheme, which attempts to homologise processes in *C. albipunctata* to *D. melanogaster*, wherever possible. Our focus lies on the early stages of development (cleavage and blastoderm stages up to the onset of gastrulation), and the formation and retraction of extraembryonic tissues. We also report the absence of visible pole cells, although expression of the germ cell marker Vasa is present at early stages.

## Results and Discussion

We characterised the development of *C. albipunctata* using time-lapse microscopy on live specimens. Selected stages were studied in more detail by imaging stained fixed embryo samples and by scanning electron microscopy.

### The life cycle of *C. albipunctata*

**Figure 1:**
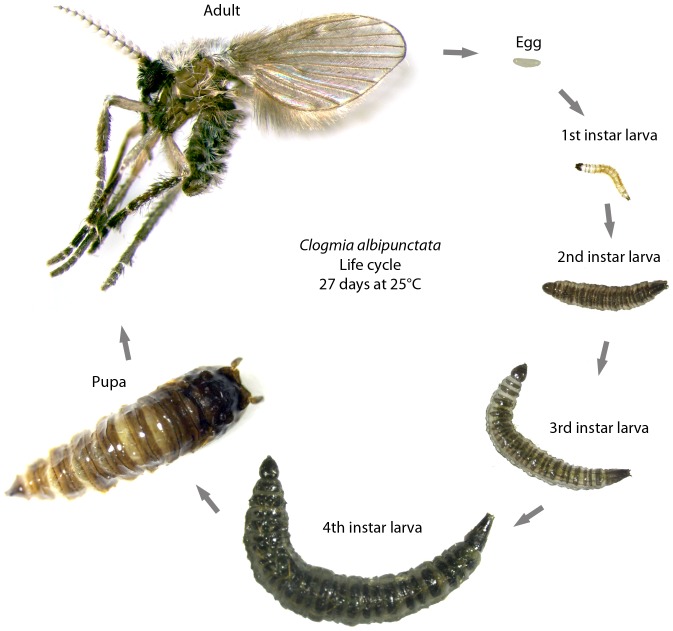
The life cycle of *C. albipunctata*. Embryonic development is covered in detail in the main text of the paper. After hatching, *C. albipunctata* goes through four larval instars before forming a pupa. The whole life cycle takes 27±5 days to complete.

Previous work suggests that the *C. albipunctata* life cycle takes about three weeks from oviposition to adult emergence at 25°C [11]. We reared *C. albipunctata* colonies at 25°C, under a 16/8 hours (hrs) day/night cycle and 75% relative humidity. Under these conditions, we observed completion of embryogenesis in approximately 3 days. We find four larval stages, each taking an average of 4.5, 3.5, 3.75 and 6.25 days respectively. The pupal stage takes about 5 days, and adults survive an average of 12 days. Adults mate shortly after emerging from the pupa, and eggs are ready to be laid 3 days after. All in all, the life cycle from adult to adult (Figure 1) takes about 27±5 days (*n* = 8), and total lifespan is about 35 days.

### Embryonic development: an overview

We produced a series of time-lapse movies using live imaging with differential interference contrast (DIC), which cover all stages of embryonic development from egg activation up to hatching of the first-instar larvae (for examples, see Movie S1 and Movie S2). Time-lapse movies were acquired at 25°C with embryos growing on a microscopy slide in water under voltalef oil. Since removal of the chorion can lead to lower survival rates and to morphological and timing defects, and since the chorion of *C. albipunctata* is transparent, imaging was performed without dechorionation. Under these conditions, embryogenesis takes approximately 3 days (71±4 h, see File S1).

In *D. melanogaster*, development can be subdivided into 17 stages (Bownes' stages) [13]. Although the overall sequence of morphogenetic events is quite conserved, there are significant differences between *C. albipunctata* and *D. melanogaster*. Because of these differences, it is not always straightforward to precisely identify homologous stages of development in each species (see below).

**Figure 2:**
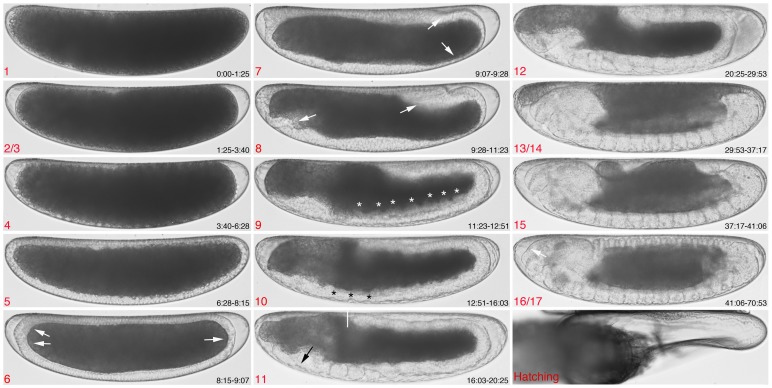
Embryonic staging and developmental events in *C. albipunctata.* Embryos are shown as lateral views: anterior is to the left, dorsal is up. Stage numbers (roughly corresponding to Bownes' stages in *D. melanogaster* [13] are shown in red at the bottom left corner of each panel. Times after egg activation from onset to end of each stage are shown in white at the bottom right corner in hrs:min. Arrows, asterisks, and white bar indicate morphological landmarks. See main text for a detailed description, and Figure 3 for comparative timing of stages with reference to *D. melanogaster*.

**Figure 3:**
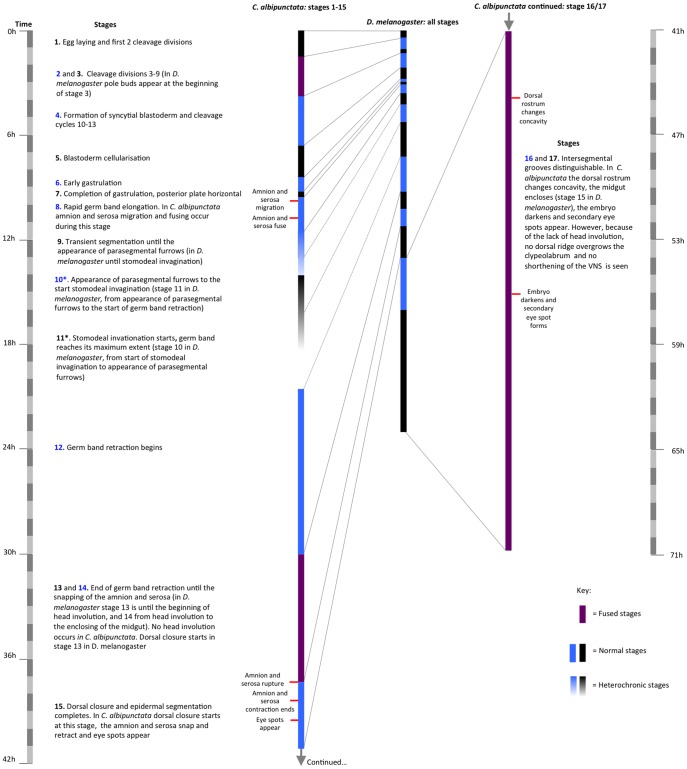
Comparative timing of stages in *C. albipunctata* and *D. melanogaster*. The duration of each stage is shown for *C. albipunctata* and *D. melanogaster* in alternating black and blue bars. Purple bars for *C. albipunctata* show fused stages due to missing landmarks. Bars with graded colours indicate hetrochronic stages. The time scale is divided into blocks of 1 hr on the far left- and right-hand side (the latter applies to *C. albipunctata* stage 16/17 shown on the right). A brief description of each stage is given. Events exclusive to *C. albipunctata* are highlighted as red crossbars.

We divide *C. albipunctata* development into 14 stages, a majority of which are homologous to equivalent stages in *D. melanogaster*. Each stage can be identified by specific morphological markers (Figure 2, and Movie S1). Since some of the defining markers for *D. melanogaster* are not present in *C. albipunctata* (i.e. formation of pole cells and head involution), we needed to fuse stages before and after the missing event (namely stages 2/3, 13/14, and 16/17; see below). Further difficulties for precise homologous staging are introduced by the fact that development in *C. albipunctata* is slower and timing more variable than in *D. melanogaster*: embryos undergo many of the same developmental events later than cyclorrhaphan flies [1], and there is much higher embryo-to-embryo variability in timing of developmental landmarks (see the large standard deviations for the different events in File S1, columns S, T, U). We have observed even higher batch-to-batch variability in our movies; embryos from the same sample differ less amongst themselves than to embryos from different experiments, carried out under the same experimental conditions at a different date (see standard deviations for siblings in File S1). Finally, we observe heterochrony in some of the *C. albipunctata* developmental landmarks compared to *D. melanogaster*. We have marked heterochronic stages (where a whole stage has shifted in time with relation to the others) with an asterisk (*) below. A comparison of *C. albipunctata* and *D. melanogaster* stages is shown in Figure 3.

In this section, we provide an overview over all stages of *C. albipunctata* development. Early development (cleavage and blastoderm stages 1–6 up to gastrulation), and extraembryonic tissue formation and retraction (stages 8–15) are described in more detail in separate sections below. All times are displayed as hrs:min unless otherwise indicated, and represent the mean of timing for ten embryos in six time-lapse movies. Raw data for the timing of each event are supplied in File S1, including the number of embryos examined (*n*), standard deviations, and the percentage of total embryonic development (% TED) for each stage (to assist identification of stages under different conditions—e.g. oil/dry—and different temperatures).

#### Stage 1

0:00–01:25 (duration: 1:25; 2% TED). Eggs are approximately 418±23 µm long and 150±17 µm wide (*n* = 140, measured with FlyGUI [14]). Stage 1 begins at egg activation. Under laboratory conditions, this is induced by a hypo-osmotic shock (see Materials & Methods). The exact time of egg activation under natural conditions remains unknown, but may occur at egg-laying when eggs suddenly encounter hypo-osmotic conditions outside the body of the mother. Stage 1 covers the first two cleavage divisions, and ends at the beginning of cleavage cycle C3. Due to the lack of visible morphological markers, we were not able to directly observe the first two cleavage cycles in any of our movies. However, we estimated the timing of these initial cycles based on fluorescent images of embryos with a DAPI nuclear counterstain, which were collected and fixed at ten-minute intervals. These data suggest that the first cleavage division occurs between 20 and 30 min, and the second between 40 and 50 min after egg activation (see File S2). We also observe that most nuclear divisions are at least slightly asynchronous, since we can frequently detect odd numbers of nuclei for some of the ten-minute sampling intervals (File S2). During this stage, the posterior pole of the embryo retracts from the chorion, leaving an empty space between the vitelline membrane and the egg cytoplasm. This happens earlier in *C. albipunctata* than in *M. abdita* [1] or *D. melanogaster* [13]. In *D. melanogaster*, stage 1 lasts for 0:25 (1.4% TED; see [13] for references to Bownes' stages during *D. melanogaster* development).

#### Stage 2/3

1:25–3:40 (duration: 2:15; 3.2% TED). In *D. melanogaster*, two different stages are defined for this period, comprising cleavage cycles C3 to C8 on the one hand (stage 2), and the formation of pole cells during C9 on the other (stage 3). Since no morphologically distinguishable pole cells are visible in *C. albipunctata* (although molecular markers indicate that the germ line is being specified at the posterior pole of the embryo; see below), we have merged those two stages into a combined stage 2/3. In *C. albipunctata*, cleavage cycles C3 to C9 occur during this stage. At some time between C5 and C6, the anterior pole of the embryo retracts from the chorion, leaving an empty space between the vitelline membrane and the egg cytoplasm. In *D. melanogaster*, the egg cytoplasm retracts from the vitelline envelope at both poles at this stage. In *C. albipunctata*, nuclei reach the periphery of the embryo during cleavage cycle C9, in contrast to *D. melanogaster*, where the blastoderm is formed a little later, one minute into C10 [15]. In *D. melanogaster*, stage 2 occurs from 0:25–1:05 and takes 0:40, and stage 3 occurs from 1:05–1:20 and lasts for 0:15 (together, 4% TED).

#### Stage 4

3:40–6:28 (duration: 2:48; 4% TED). Stage 4 begins when the nuclei have reached the periphery of the embryo at the end of C9 to form the syncytial blastoderm. This stage includes cleavage cycles C10 to C13. At some time during C10 and C11, the space between the vitelline membrane and the egg is refilled, first at the anterior, then at the posterior pole. This stage terminates at the beginning of cleavage cycle C14. During each of the cleavage divisions (except at C10), we observe the ingression of membranes, which is reversed when the following cleavage cycle begins (see below). *D. melanogaster* forms similar abortive membrane invaginations called metaphase (or pseudo-cleavage) furrows. In *D. melanogaster*, this stage occurs from 1:20–2:10 and lasts for 0:50 (3.5% TED).

#### Stage 5

6:28–8:15 (duration: 1:47; 2.5% TED). Stage 5 lasts from the beginning of cleavage cycle C14 to the start of gastrulation. During this stage, cellular membranes form, which progressively surround the blastoderm nuclei. Nuclear morphology changes from circular to elongated (see below). Stage 5 ends as the ventral blastoderm cells start to exhibit a wavy appearance (seen as uneven apical and basal surfaces), which marks the onset of gastrulation. In *D. melanogaster*, this stage occurs from 2:10–2:50 and lasts for 0:40 (3% TED).

#### Stage 6

8:15–9:07 (duration: 0:52; 1.2% TED). Gastrulation happens at this stage. The first sign of gastrulation is a wavy appearance of the ventral cells, and the thickening of the medio-ventral periplasm. Ventral cells migrate laterally and upwards. This is followed by the flattening of the anterior-most blastoderm cells, thickening of the anterio-dorsal tip of the embryo, and the formation and dorsal shift of the posterior plate—which, in contrast to *D. melanogaster* [13] and *M. abdita* [1], does not contain morphologically differentiated pole cells (Figure 2, stage 6, white arrows). This is followed by an upward movement of the cells at the anterior and posterior poles. During this process, the postero-ventral cells become thinner. In the anterior, a flattening of the dorsal-most cells occurs, a process which will later be important for the migration of the extraembryonic tissues (at stage 8). In *D. melanogaster*, the ventral and cephalic furrows form at this stage. Surprisingly, we could not observe the formation of any clearly visible furrows in our movies of *C. albipunctata*. This is likely to be at least partially an artifact of the imaging methodology, since we observe a ventral furrow in scanning electron micrographs (not shown). However, we never observe embryos with a cephalic furrow suggesting that this morphological feature is absent in *C. albipunctata*. In *D. melanogaster*, this stage occurs from 2:50–3:00 and lasts for 0:10 (1% TED).

#### Stage 7

9:07–9:28 (duration: 0:21; 0.5% TED). Stage 7 begins with the posterior plate (which does not carry morphologically distinctive pole cells) in a horizontal position (parallel to the A–P axis), and the end of the thinning of the postero-ventral region (Figure 2, stage 7, white arrows). The posterior plate—which behaves like the cell plate bearing the pole cells in *D. melanogaster* and *M. abdita*—continues to tilt forming a pocket (the amnioproctodeal invagination). Initiation of cephalad (headwards) movement of this plate marks the end of stage 7. In *D. melanogaster*, the dorsal folds become visible at this stage, but we do not observe them in our movies. In *D. melanogaster*, this stage occurs from 3:00–3:10 and lasts for 0:10 (1% TED).

#### Stage 8

9:28–11:23 (duration: 1:55; 2.7% TED). This stage starts with the initiation of the rapid phase of germband extension, and the associated anterior movement of the amnioproctodeal invagination. The rapid extension of the germband takes place at this stage. The germband reaches approximately 55–60% A–P position (where 0% is the anterior pole). The amnioserosal lip forms (Figure 2, stage 8, posterior white arrow), from which the tissue fold formed by amnion and serosa starts to migrate ventrally. As the amnion and serosa migrate to the anterior pole, cells in the ventral head region invaginate (Figure 2, stage 8, anterior white arrow), leaving a thickening at this position. Migration of the amnion and serosa around the embryo finishes when the two migrating folds fuse at the ventral side of the embryo (see below). After this fusion, we observe a membrane growing towards the two poles of the embryo, a process which might mark the end of extraembryonic tissue formation (see Movie S1). In *M. abdita*, the serosa starts to migrate at stage 8, but migration lasts much longer, until stage 11 [1]. In *D. melanogaster*, this stage occurs from 3:10–3:40 (2% TED), during which the germband reaches more than 40% A–P position, but no migration of extraembryonic tissues occurs.

#### Stage 9

11:23–12:51 (duration: 1:28; 2% TED). Stage 9 begins with the formation of transient mesodermal segmentation (Figure 2, stage 9, asterisks). The germband continues to extend (starting the slow phase of germband extension, which will finish at stage 11). In *D. melanogaster*, this stage happens from 3:40–4:20, and lasts for 0:40 (3% TED).

#### Stage 10*

12:51–16:03 (duration: 3:12; 4.5% TED). Stage 10* begins with the appearance of parasegmental furrows (Figure 2, stage 10, asterisks). The germband continues to slowly extend. This constitutes a clear heterochrony between *D. melanogaster*/*M. abdita* and *C. albipunctata*, since parasegmental furrows only appear at stage 11 in the former two species. Consequently, stage 10* in *C. albipunctata* cannot be homologised to stage 10 in *D. melanogaster*, but is more likely to be homologous to stage 11. That stage occurs from 5:20–7:20 in *D. melanogaster* and lasts for 2:00 (8% TED).

#### Stage 11*

16:03–20:25 (duration: 4:22; 6.2% TED). At the beginning of this stage the stomodeal invagination starts forming (Figure 2, stage 11, black arrow). The germband reaches its maximum extent, around 35% A–P position, and stays extended during the whole stage (Figure 2, stage 11, white bar). In *D. melanogaster*, the germband reaches its maximum extent, at 25% A–P length, at stage 10, which occurs from 4:20–5:20, and lasts for 1:00 (4% TED), before the parasegmental furrows appear. Therefore, stage 11* in *C. albipunctata* is likely to be homologous to stage 10 in *D. melanogaster*.

#### Stage 12

20:25–29:53 (duration: 9:28; 13.4% TED). This stage starts with the beginning of germband retraction, which is completed at the start of stage 13/14. In *D. melanogaster*, germband retraction occurs from 7:20–9:20, and lasts for 2:00 (8% TED).

#### Stage 13/14

29:53–37:17 (duration: 7:24; 10.4% TED). This stage starts with the completion of germband retraction, and lasts until just before the extraembryonic tissues rupture at a ventral position. *D. melanogaster* is not protected by fully formed extraembryonic membranes. Only its dorsal opening is covered by a reduced and fused amnioserosa. In contrast, in *C. albipunctata*, the entire surface of the embryo remains surrounded by a double-layered extraembryonic structure formed by the amnion and the serosa at this time of development. In *D. melanogaster* and *M. abdita*, head involution begins at stage 14. This process does not occur in nematocerans such as *C. albipunctata*, where larvae retain external gnathal and procephalic head tissues with their associated sense organs and head skeletal structures. Since there is no head involution in *C. albipunctata*, we have fused stages 13 and 14 into a combined stage 13/14. In *D. melanogaster*, stage 13 occurs from 9:20–10:20, while stage 14 occurs from 10:20–11:20. Both stages last 1:00 each (together, 8% TED).

#### Stage 15

37:17–41:06 (duration: 3:49; 5.4% TED). Stage 15 starts when the amnion and the serosa rupture at a ventro-medial position. These membranes retract towards the dorsal opening (which takes a larger portion of the A–P axis than in *D. melanogaster*, since the head in *C. albipunctata* is smaller) rounding the anterior pole before the posterior. This retraction process takes about 1:00. Dorsal closure starts when the aminioserosal retraction finishes. Segmentation of the dorsal epidermis is also completed at this stage, and the larval eye spots become visible. In *D. melanogaster*, the gut constricts during this stage, but we do not observe this process in *C. albipunctata*. This is not surprising since gut constriction is associated with head involution. In *D. melanogaster*, stage 15 occurs from 11:20–13:00 and lasts for 1:40 (7% TED), starting with the closure of the midgut, which in *C. albipunctata* happens at stage 16/17.

#### Stage 16/17

41:06–70:53 (duration: 29:47; 42% TED). Stage 16/17 begins with the appearance of the intersegmental grooves at the mid-dorsal of the embryo. In *D. melanogaster*, this conincides with the shortening of the ventral nervous system (VNS). *C. albipunctata* does not seem to go through this shortening process. Stage 17 of *D. melanogaster* starts with the dorsal ridge overgrowing the clypeolabrum. Due to the lack of head involution, we do not observe this process in *C. albipunctata*. For this reason, we have fused stages 16 and 17 into a combined stage 16/17. We do observe, however, that the dorsal part of the rostrum changes concavity while the *C. albipunctata* larva prepares to hatch, the head getting closer to the chorion during this process (Figure 2, stage 16/17, white arrow). About 15 hrs before hatching, the embryo substantially darkens and the eye spot differentiates forming a secondary domain. We observe the onset of muscle contractions only 7 hrs before hatching, much later than in *D. melanogaster* or *M. abdita* (where contractions already happen at stage 15). We are unable to see the trachea filling with air in our movies. The first instar larva hatches at around three days after egg activation (71±4 h). In *D. melanogaster*, stage 16 takes 3:00 (13% TED), from 13:00 to 16:00, and stage 17 occurs from 16:00–24:00 and lasts for 8:00 (33% TED).

### Detailed staging of early embryogenesis in *C. albipunctata*

**C. albipunctata has 14 cleavage cycles.** So far, almost all experimental studies using C. albipunctata have focussed on early stages of development [7]–[11]. Because of this, we decided to characterise the pre-gastrulation development of C. albipunctata in more detail. We focus on the cleavage and blastoderm stages. Using time-lapse movies, we can observe cleavage divisions by tracking the disintegration and reappearance of nuclear envelopes from cleavage cycle C3 onwards (see Movie S1 and Movie S2). Earlier cleavage divisions are difficult to observe directly using DIC live imaging due to the lack of clearly visible morphogenetic landmarks (see previous section).

**Figure 4:**
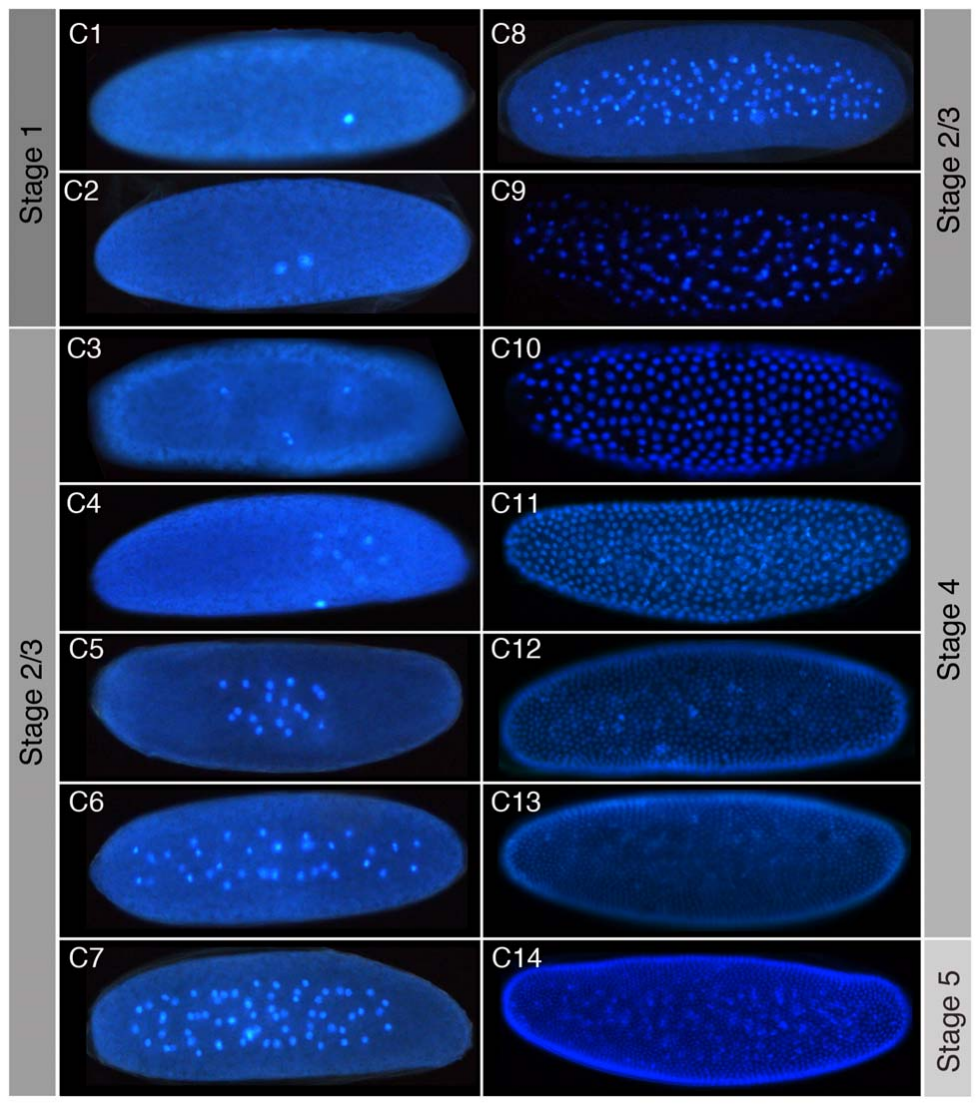
Cleavage cycles of *C. albipunctata*. Fluorescence images of embryos with DAPI-counterstained nuclei are shown as lateral views. Anterior is to the left. C1–14 indicates cleavage cycle number. The focus is on the sagittal plane for embryos at cleavage stage (C1–C9), and lateral views are shown at blastoderm stage (C10–14). As in *D. melanogaster*, nuclei begin to move towards the periphery from C7 onwards. Corresponding embryonic stages (see Figures 2 and 3) are indicated on grey background.

For this reason—and to confirm our live imaging analysis by an independent approach—we used heat-fixed embryos with a DAPI nuclear counterstain to examine the number of pre-gastrulation cleavage divisions. Nuclear counts based on fluorescent images allow us to establish the number of cleavage divisions (Figure 4, Table 1; File S2; in addition, we provide scanning electron micrographs of C11–14 in File S3). Up until cleavage cycle C8, we find embryos with nuclear counts that are very close to the theoretically expected figure (2^n^, where *n* is the number of preceding cleavage divisions). We also see, as described in the previous section, that cleavage divisions show a lesser degree of synchronicity than in *D. melanogaster* [15] or *M. abdita* [1]. At C8, nuclei start to migrate to the periphery to form the blastoderm layer at the surface of the embryo. Once the blastoderm is formed (from late cleavage cycle C9 onwards), we can only focus on one side of the embryo, and therefore only count half of the nuclei in each embryo. An additional amount of nuclei will be missed due to overlap and loss of focus at the edges of an embryo image (especially from C11 onwards). Still, based on nuclear counts, embryos can be grouped into clearly distinguishable classes corresponding to individual cleavage cycles (Table 1). Our data strongly suggest that there are 14 cleavage cycles in *C. albipunctata*, the same number as in *D. melanogaster* [15], and *M. abdita* [1].

**Table 1:** Observed number of nuclei in *C. albipunctata*.

Cleavage Cycle	Expected # of Nuclei	*C. albipunctata* Nuclei Count
C1	1	1±0 (n = 14)
C2	2	2±0 (n = 15)
C3	4	3.5±0.51 (n = 19)
C4	8	6.2±0.9 (n = 21)
C5	16	14.1±2.5 (n = 13)
C6	32	30.5±1.8 (n = 20)
C7	64	62 (n = 1)
C8	128	105±4.9 (n = 7)
C9	256 (nuclei start to migrate)	161 (n = 1)
C10	512	247.5±28 (n = 4)
C11	1024	345.7±39.7 (n = 23)
C12	2048	711.4±51.5 (n = 5)
C13	4096	979.3±44.9 (n = 7)
C14	8192	1317.7±201.7 (n = 6)

Number counts (with standard deviations) based on *n* DAPI-stained embryos (such as those shown in Figure 4) are compared to those expected considering preceding mitotic divisions. Note that expected numbers are overestimates from C9 onward, as nuclei migrate out of the plane of focus and some remain behind in the yolk (also outside the focal plane).

**Length and subdivision of blastoderm cycles.** The main focus of our work is quantification and mathematical modelling of segmentation gene expression (see, for example, [16]–[25]). For the purpose of embryo staging and quantitative simulation, we need a high-resolution staging scheme for the blastoderm stage in C. albipunctata. Therefore, we carefully characterise the precise timing of cleavage cycles C10–C14A (the portion of C14 that occurs before gastrulation). Because C14A is considerably longer than the other blastoderm cycles, it has been subdivided into 8 time classes in D. melanogaster [20], [26]. In order to facilitate cross-species comparisons of gene expression patterns, we also divide C14A in C. albipunctata into 8 time classes.

**Figure 5:**
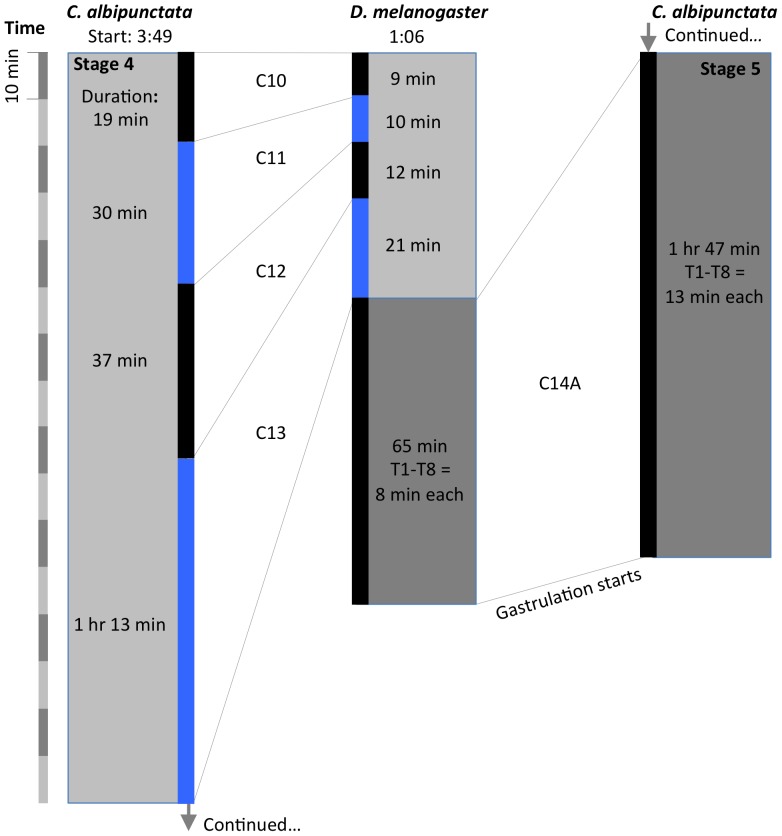
Comparison of the length of blastoderm cycles in *D. melanogaster* and *C. albipunctata*. The duration of each division cycle is shown for both species with alternating black and blue bars. The onset of each cycle corresponds to the reappearance of nuclear envelopes in DIC movies. The time scale on the left is divided into blocks of 10(in hrs:min after egg activation) and duration (in min) are shown for cleavage cycles C10–14. For *D. melanogaster*, time for the start of C10 is taken from [13], and times for the duration of the blastoderm cycles from [15]. Corresponding embryonic stages (see Figures 2 and 3) are indicated on grey background.

We have used DIC time-lapse movies (see Movie S2) to describe the timing and duration of each of the blastoderm cycles as described above (Figure 5). In *D. melanogaster*, C10 to C14A last for 9, 10, 12, 21, and 65 min respectively [15], [27]. In *C. albipunctata*, the corresponding times are 19, 30, 37, 73, and 107 min.

In addition to establishing the timing and duration of blastoderm cleavage cycles, we have analysed membrane morphology and nuclear shape at each of the cleavage cycles C10–13, as well as eight evenly-spaced time classes (T1–8) during C14A. This scheme can be used to stage blastoderm embryos at a high temporal resolution of down to about 13 min.

**Figure 6:**
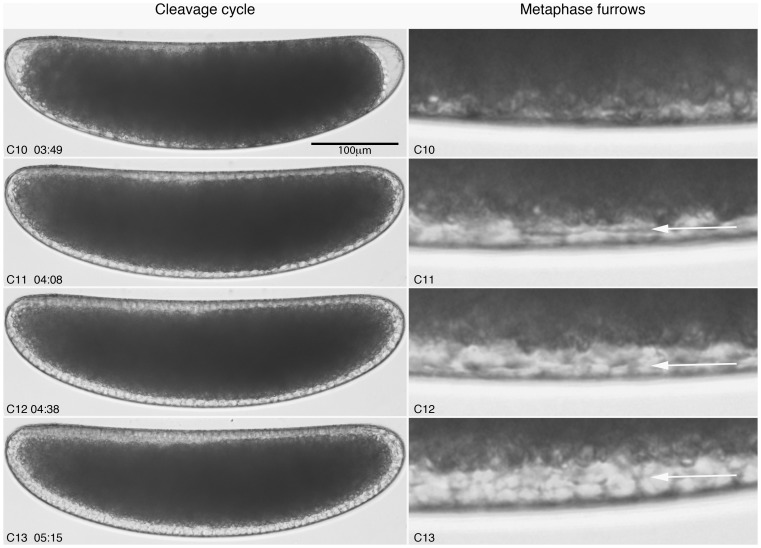
*C.albipunctata e*arly blastoderm cycles (C10–13). Captured images from live DIC movies. Images show lateral views, anterior is to the left. Images on the right show metaphase (pseudo-cleavage) furrows (membrane progression marked by white arrows). Note that we cannot see metaphase furrows at C10. Times indicate time of cycle start in hrs:min after egg activation.

**Figure 7:**
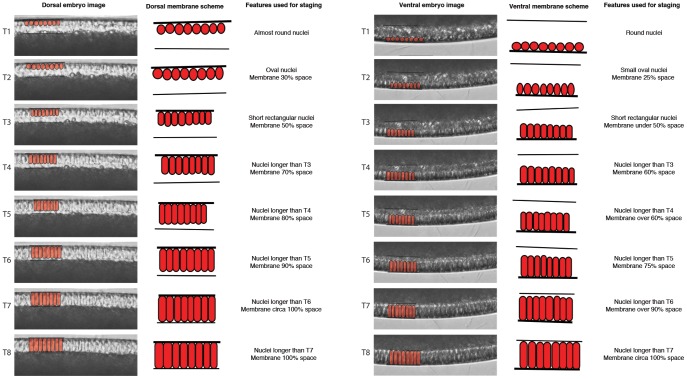
Cellularisation and time classification scheme for *C. albipunctata* during cleavage cycle C14A. Images captured from time-lapse movies showing the membrane morphology at mid-dorsal (left column) and mid-ventral (right column) positions are shown on the left-hand side of each column for time classes T1–T8. Schematic overlays in the middle of each column show vitelline membrane (thick black line), nuclei (red circle, oval or rectangle), and the edge of the periplasm, where the yolk begins (thin black line). Text on the right-hand side represents descriptions of features used to characterise each stage. See text for details.

During C10 to C13, we observe the expected increase in nuclear number and density. The round shape of nuclei remains relatively constant throughout this period. We observe the invagination of metaphase (or pseudo-cleavage) furrows at every cycle from C11, but membrane progression is aborted as the new cycle starts (Figure 6; see also [28], [29]). At the beginning of C14, definitive cellularisation begins as membranes invaginate and increasingly surround the blastoderm nuclei. During the progression of the membrane front from the apical to the basal side of the nuclei, nuclear shapes change from round to elongated (Figure 7). In contrast to other species [1], [15], we observe only subtle morphological differences between stages when using mid-dorsal regions only. To make our staging system more robust, we therefore include a mid-ventral region into our scheme (Figure 7). Both dorsal and ventral regions go through the same sequence of membrane front progression and nuclear shape changes, but progress is slightly faster dorsally than ventrally. At time class 1 (T1), nuclei are round ventrally and slightly oval dorsally. No membrane is visible yet. By T2, nuclei are oval (slightly smaller ventrally) and the membrane has progressed to cover about 30% of the periplasmic space dorsally, and 25% on the ventral side. From this point, the membrane front progresses together with the basal edge of the extending nuclei. At T3, nuclei become increasingly elongated, and the membrane takes up approximately 50% of the periplasm dorsally (slightly less ventrally). From here on, nuclei are elongating progressively at every subsequent stage. At T4, the membrane and nuclei take up about 70% of the periplasm on the dorsal side of the embryo, and about 60% at ventrally. At T5, the membrane is at about 80% dorsally, and just under 70% on the ventral side. At T6, the membrane is filling approximately 90% of the periplasm dorsally, and about 75% on the ventral side. At T7, membranes and nuclei take up almost all of the available space in the periplasm on the dorsal side, while ventrally, membranes and nuclei reach over 90%. At T8, dorsal membranes reach the yolk, filling the entire yolk-free periplasm, while on the ventral side a small proportion of membrane-free periplasm remains basally. T8 ends with the onset of gastrulation.

#### Staging gene expression: *even-skipped* in the blastoderm

In *D. melanogaster*, the pair-rule gene *even-skipped* (*eve*) shows a very dynamic expression pattern in the blastoderm, and has been used as a marker for the precise staging of embryos [17], [19], [20], [26]. Here, we demonstrate the utility of our staging system, by applying it to a detailed analysis of the blastoderm-stage expression of one of the two *eve* paralogues, the *eve1* gene [10], in *C. albipunctata*. Both *eve* paralogues show identical expression patterns at the blastoderm stage [11]. For simplicity, we will refer to *eve1* as *eve* in what follows.

Previous characterisations of the *C. albipunctata eve* RNA expression pattern either used only one time point at blastoderm stage [8], or was based on a staging scheme that used the time that elapsed between induced egg activation by osmotic shock and embryo fixation [11]. This can lead to inaccuracies due to the large embryo-to-embryo variability in developmental timing (see above). As a more accurate alternative, we have produced a time series of *eve* expression according to our morphological classification scheme (see previous section). We find some artefacts in nuclear morphology that may be related to the fixation conditions. However, the relationship between the size of the nuclei and the yolk free periplasmic space remains consistent allowing classification of these embryos.

**Figure 8:**
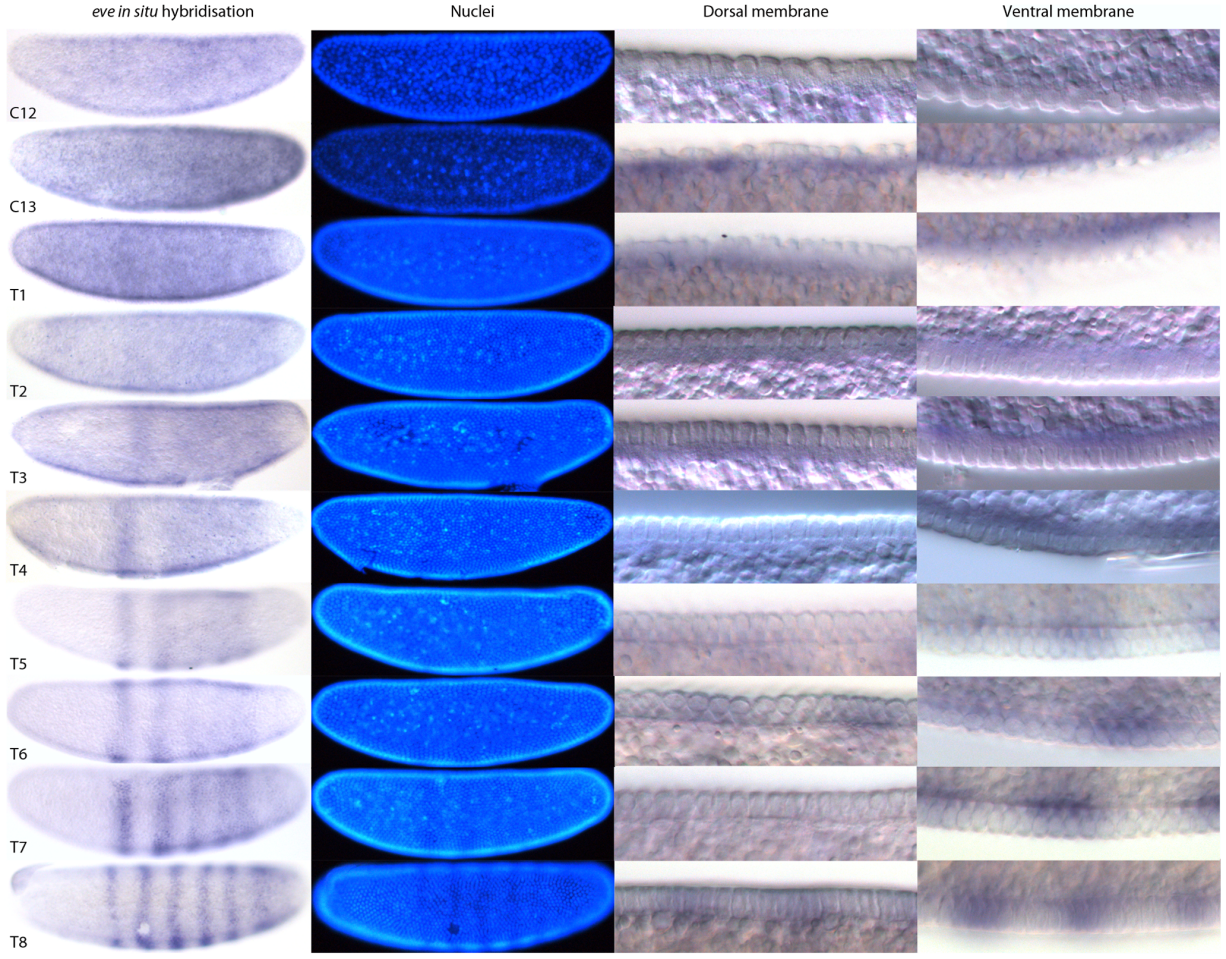
*C. albipunctata eve* expression staged using nuclei number, nuclear density, and membrane morphology. Our staging method first distinguishes cleavage cycles based on the number of nuclei observed. Membrane morphology is then used to check the assignment of embryos to cleavage cycles C10–13 based on the size and spacing of the nuclei (see Figure 6). Embryos assigned to cleavage cycle C14A are further classified into time classes T1–8 based on membrane morphology and nuclear shape (see Figure 7). Using this method, we provide a detailed time-series for expression of the pair-rule gene *even-skipped* (*eve*) during the blastoderm stage. Lateral views are shown: enzymatic *in situ* hybridisation stains to the left, and DAPI-counterstain in the middle. The two columns on the right show details of dorsal (left) and ventral (right) membrane/nuclear morphology (sagittal views). See text for details.

For each time class between C12 and C14A-T8 in *C. albipunctata*, we acquired brightfield and DIC images of whole embryos stained by *in situ* hybridisation against *eve* mRNA, plus higher-magnification DIC images with details of dorsal and ventral membrane morphology (as described in [14]) (Figure 8). In addition, embryos were counterstained against nuclei using DAPI. Time classification was carried out independently by two researchers according to nuclear count, shape, and membrane morphology as described in the previous section.

We first detect *C. albipunctata eve* expression during C12 from around 13 to 100% A–P position (Figure 8). This expression expands further into the anterior during C13 and T1. At T1, expression retracts slightly from the posterior pole leaving an enlarged non-expressing region covering around 94–100% A–P position. Through T2 and T3, expression decreases in the anterior until at T3 stripe 1 appears at around 38–48% A–P position. Posterior to stripe 1, another region of expression begins to clear, separating a large posterior expression domain at 54–94% A-P position. At T4, stripe 1 has separated from the large posterior domain. At T5 stripe 2 begins to form from the anterior border of the posterior domain at around 50–55% A–P position but remains part of the larger posterior domain that has retracted to 90% A–P position. At T6 stripe 2 has separated from the posterior domain, and stripe 3 has started to form from the posterior domain that has retracted further to around 85% A–P position. In this posterior space, weak expression of stripe 6 appears. At T7 stripe 3 has separated and the posterior domain—now at around 68–85% A–P position—has begun to resolve into stripes 4 and 5. In the meantime, stripe 6 is intensifying at 88–94% A–P position. By T8, each of the six pre-gastrulation *eve* stripes have formed and separated.

In summary, we find that the dynamics of *eve* expression is generally shifted towards the end of C14A in *C. albipunctata* compared to *M. abdita* [1] and *D. melanogaster* [20], where all 7 stripes can be distinguished by T4. This is consistent with a quantitative analysis at the protein level [19], and extends and confirms results from previous studies, which revealed the presence of a heterochronic shift in the posterior, as *eve* stripe 7 does not form until after gastrulation [8], [11].

### Extraembryonic tissues in *C. albipunctata*

The extraembryonic tissues (amnion and serosa) of dipterans are vital in their early development, due to their involvement in axis formation and patterning of the early embryo [30]. In *D. melanogaster*, both tissues have been reduced and fused to form a dorsal amnioserosa. In contrast, both tissues are present as fully developed membranes in *C. albipunctata* [31], [32].

**Figure 9:**
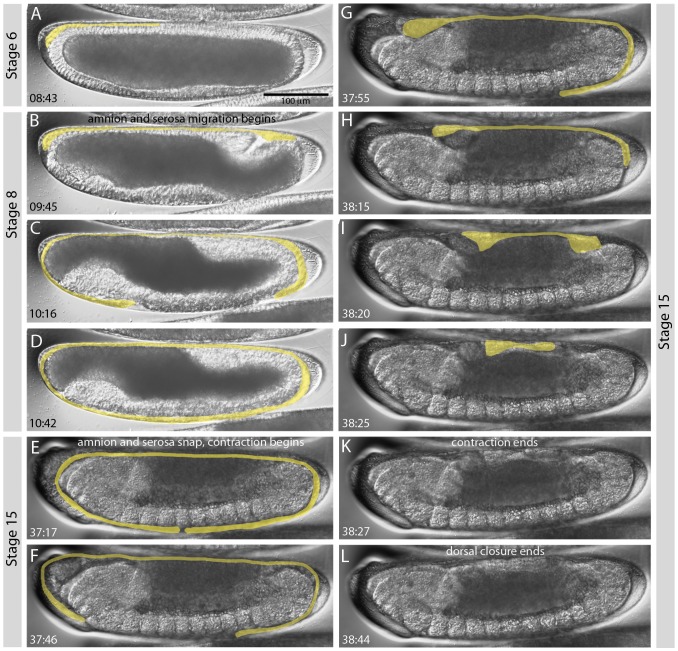
Extension and retraction of extraembryonic tissues in *C. albipunctata*. Time is shown in hrs:min after egg activation for each image. Extraembryonic tissues are highlighted in yellow. Corresponding embryonic stages (see Figures 2 and 3) are indicated on grey background. See text for details.

We have characterised the stages of extraembryonic tissue formation, extension, and retraction in *C. albipunctata*. The amnion and serosa start forming just after gastrulation, at stage 6, when a thickening at the antero-dorsal part of the embryo appears (Figure 9A). At stage 8, the amnio-serosal lip appears as the cells that form the posterior plate ingress at the posterio-dorsal side of the embryo (Figure 9B). From this lip, an extraembryonic fold moves posteriorly and ventrally, while the antero-dorsal thickening starts to slowly migrate anteriorly and ventrally, both following the surface of the embryo (Figure 9C). These folds fuse at a medio-ventral location, completely enveloping the embryo with two layers—serosal, on the outer side and amniotic below that—at the end of stage 8 (Figure 9D). The two layers are not clearly distinguishable in our movies, but can be inferred from the presence of the migrating folds. Finally, we observe a membrane growing towards the two poles of the embryo just after this fusion. This process may contribute to the completion of extraembryonic tissue formation. Amnion and serosa completely surround the embryo throughout extension and retraction of the germband until stage 15, when they rupture from the ventral side (Figure 9E). Both tissues then rapidly retract towards the dorsal opening of the embryo (Figure 9F), first reaching the anterior pole of the embryo (Figure 9G), then the posterior (Figure 9H). The whole retraction process to the end of the contraction takes about 70 min (Figure 9E–K). When the retracting tissues reach the dorsal opening, they are reabsorbed. This process seems to be tightly linked to dorsal closure, which finishes 20 min after retraction of extraembryonic tissues is complete (Figure 9L).

### Germ line development: formation of the pole cells

The germline of *D. melanogaster* separates from the somatic cells early in development. This can be observed through the formation of pole buds at stage 3. These pole buds divide once during this stage, and once more at stage 4, before pinching off to form 12–14 pole cells. A second division results in a total of 34–37 pole cells.

**Figure 10:**
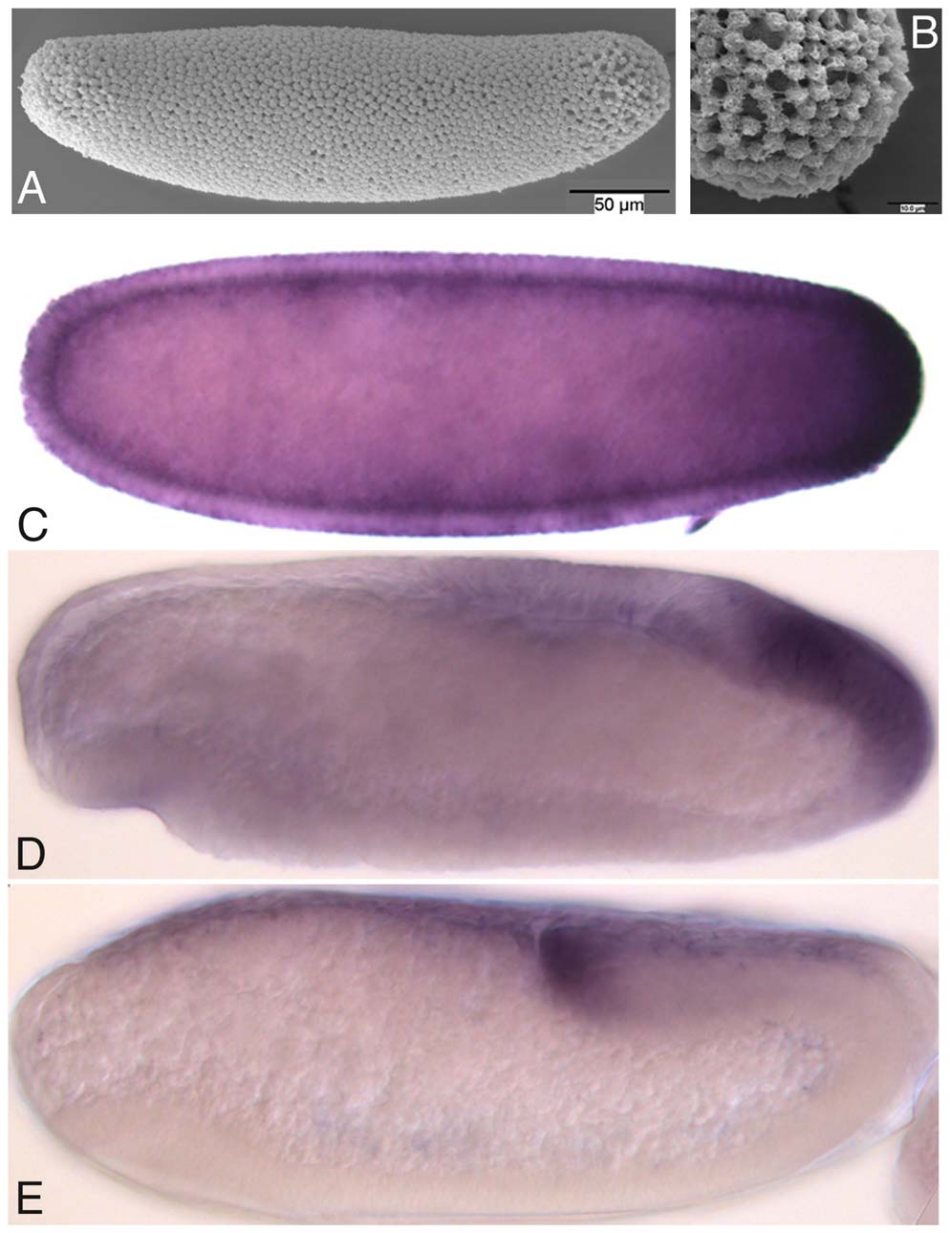
Germ plasm in *C. albipunctata*. (A) Scanning electron micrograph of a stage 4 embryo showing the absence of morphologically distinguishable pole cells. (B) Close up of the posterior pole of the embryo shown in (A). (C–E) Vasa antibody stains of embryos at stage 4 (C), 6 (D), and 8 (E). Lateral views, anterior is to the left, dorsal is up. See text for details.

We could not identify morphologically differentiated pole buds or pole cells in *C. albipunctata* (Figure 10A, B). We have used the highly conserved germline marker protein Vasa [33] to check for morphologically undifferentiated cells that could be the germ cell precursors. We identify a posterior region in the embryo—corresponding to where the pole cells are in *D. melanogaster*—which shows Vasa protein expression (Figure 10C–E). We first detect Vasa at stage 4 (Figure 10C), and its expression persists until germband extension (Figure 10D, E). Vasa could be expressed even earlier, since we cannot process embryos earlier than stage 4 for immunostaining. This evidence suggests that, although we cannot morphologically differentiate pole cells, *C. albipunctata* does have germ line precursors showing Vasa expression at the same position as *D. melanogaster*.

## Conclusion

This paper presents a detailed and systematic description of the life cycle and embryonic development of the moth midge *C. albipunctata*, while the accompanying paper by Wotton *et al.* [1] provides an equivalent characterisation for the scuttle fly *Megaselia abdita*. These two papers provide a useful resource and reference for the growing community of fly geneticists and evolutionary developmental biologists studying non-drosophilid dipteran species.

We propose an embryonic staging scheme (Figures 2 and 3), which is as homologous as possible to the one already established for *D. melanogaster* [13]. This scheme covers the entire duration of embryonic development. In addition, we have examined specific processes and stages of development in more detail. We establish that *C. albipunctata* has 14 cleavage divisions (Figure 4), and precisely measure the timing and length of blastoderm division cycles (Figure 5). Based on this, we propose a detailed staging scheme for these early stages of development using membrane morphology and nuclear number/shape as morphological markers (Figures 6 and 7). We use this staging scheme to provide a careful analysis of *eve* expression in the *C. albipunctata* blastoderm (Figure 8). In addition, we characterise the extension and retraction of extraembryonic tissues (Figure 9). Finally, we demonstrate that, despite the lack of morphologically differentiated pole cells, *C. albipunctata* germ cells appear to form in the posterior of the blastoderm-stage embryo, similar to those in *D. melanogaster* [13] and *M. abdita* [1].

Considering the large evolutionary distance between a nematoceran species, such as *C. albipuncatata*, and cyclorrhaphan brachycerans, such as *M. abdita* and *D. melanogaster*, the overall dynamics and morphology of development is remarkably conserved in all dipterans examined so far. Many landmark processes, such as cleavage, cellularisation, gastrulation, germband extension and retraction, mesoderm and midgut formation, and even dorsal closure are similar and occur at comparable stages in development. This enables us to homologise many developmental processes across the entire order of Diptera.

Despite this large degree of overall conservation, we detect a number of interesting and important differences between *C. albipuncatata* and other dipteran species. As mentioned above, *C. albipunctata* shows no overtly differentiated pole buds or pole cells. Other characteristic traits such as the head furrow or dorsal folds are missing, as are landmark developmental processes such as head involution, gut constriction, and the shortening of the VNS. Other processes show clear morphological differences between species. Extraembryonic tissues are fully developed in *C. albipunctata*, where folds consisting of amnion and serosa extend to surround the entire embryo. In contrast, the amnion is restricted to the dorsal side of the embryo in *M. abdita* [1], [32], and *D. melanogaster* only shows a strongly reduced and fused dorsal amnioserosa. Interesting morphological differences can also be observed in gastrulation. While mosquitoes—such as the malaria vector *Anopheles gambiae*—have no ventral furrow, but show ingression of individual mesodermal cells instead [34], we detect invagination through ventral furrow formation in scanning electron micrographs of *C. albipunctata*. In this regard, *C. albipunctata* is more similar to cyclorrhaphan species such as *M. abdita* and *D. melanogaster*. Finally, we observe heterochronic shifts in some developmental features, such as the delayed appearance of *eve* stripes, or the premature formation of parasegmental furrows.

These significant differences in embryogenetic processes between *C. albipunctata* and both mosquitoes and brachycerans, suggest that many morphological changes in dipteran development must have occurred during the early radiations of the Diptera. In contrast, the extremely conserved nature of development among cyclorrhaphan species such as *M. abdita* and *D. melanogaster* [1] seems to indicate a slower rate of morphological evolution within that group.

## Materials and Methods

### Fly culture and embryo collection

*C. albipunctata* fly cultures were kept as described in [11], with the following modifications: the animals were fed on dusted spirulina-based fish food, and the day/night time cycle was set to 16/8 hrs. Eggs were dissected from female adults, activated by osmotic shock, and (for fixed samples) embryos were dechorionated as described in [11]. Embryos for DAPI staining, *in situ* hybridisation, and immunostaining were heat-fixed using a protocol adapted from [35]. To image live specimens, we put embryos on a slide with a small amount of water and cover them with 10S voltalef oil but no coverslip. Imaging typically started 10–20 min after egg activation by osmotic shock.

### Life cycle imaging

Adult and larval stage images for Figure 1 were captured using a Leica EC3 camera mounted on a dissecting stereoscope. A light diffuser consisting of a cylinder of white paper was used to spread light from the light source evenly over a sample mounted on a glass needle. Multiple *z*-stacks of each sample were taken and in-focus regions patched together using Photoshop.

### Embryo imaging

Embryo images for Figures 4, 8, and 10 were taken using a Leica DM6000B upright compound microscope using a 10× objective. Pictures for DAPI counterstaining, *in situ* hybridisation, and antibody staining experiments were acquired and processed as described in [21].

### Time-lapse imaging

Slides were placed on a temperature-controlled platform at 25°C. Embryos were imaged with a Leica DM6000B upright compound microscope using 10× or 20× objectives, and time intervals between image acquisitions every 1 min. Specifications of optics, magnification, time interval, and embryo orientation for each time-lapse are provided in File S1. Movies were processed using ImageJ (http://rsbweb.nih.gov/ij).

### Nuclear staining

Counterstaining with DAPI was performed as follows: fixed methanol-dehydrated embryos were rehydrated into PBT and incubated with 0.3 µM DAPI in PBT for 10 minutes. The embryos were then washed 3× in PBT followed by 3×10 min in PBT, and cleared by a dilution series of 30/50/70% glycerol/PBS. Stained embryos were mounted and stored in 70% glycerol/PBS.

### *In Situ* Hybridisation

*In situ* hybridisation was performed as described in [4].

### Antibody Staining

Vasa immunostainings were performed using an antibody kindly provided by P. Lasko at a dilution of 1:250. Fixed embryos, stored in methanol (see previous section), were rehydrated for 5 min in PBT/Methanol (1/1) and washed in PBT (2×1 min, 1×20 min) at room temperature (as were all subsequent stages unless indicated). Blocking was carried out with 2×30 min washes in PBT with Western Blocking Reagent (PBTB) (Roche). Incubation with primary antibody was in PBTB for 3 hrs. 3× PBT washes were performed followed by a final overnight wash in PBT at 4°C. Blocking for the secondary antibody was performed as described previously. Incubation with secondary antibody (goat anti-rabbit, 1:3000; Jackson ImmunoResearch Laboratories, Inc.) was carried out in PBTB for 1 hr. Washes were 3× in PBT and 4×15 min in PBT. Pre-stain washes were 2×5 min in AP Buffer (100 mM NaCl, 50 mM MgCl_2_, 100 mM Tris PH 9.5, 0.1% Tween). Staining was performed in AP Buffer with 1 µl/ml NBT and BCIP (Roche). After staining, embryos were washed in PBT, followed by DAPI staining and mounting as described above.

### Scanning Electron Microscopy

Scanning images were taken with a Zeiss DSM 940A scanning electron microscope at the Unitat de Microscopia Electronica (Campus Casanova) of the University of Barcelona. Samples were processed as follows: samples were fixed using 2.5% glutaraldehyde in 0.1 M cacodylate buffer overnight at 4°C, followed by 3×10 min washes at 4°C in 0.1 M cacodylate buffer. Post-fixation was performed in 1% osmium tetroxide in 0.1 M cacodylate buffer for 2 h at 4°C followed by 3×10 min washes at 4°C in milliQ water. Embryos were put through an ethanol dilution series of 25, 50 and 70%, each for 10 min at 4°C, then 3×10 min additional washes in 90, 96 and 100% ethanol at 4°C. Embryos were critical-point-dried using a VGMicrotech CPD 7501 system, and gold coating was carried out using a Fisons Instrument FC510 Sputtering System.

## Supporting Information

File S1 **Timing of developmental events from individual time-lapse movies in** ***C. albipunctata.*** Stages and developmental events are shown in columns A and B. Time-lapse (TL) movie IDs are listed along the top, with embryo numerical ID, along with averages of timing of events across embryos/movies in minutes (min) and hours:minutes (hh:mm). Also listed are the number of embryos *n* underlying the calculation of average times for each event, stage duration (in hh:mm), standard deviation (st deviation, in min) for each event. % of developmental time is also shown for each stage. Rows 4–6 detail the optics (10×, or 20×), the embryo view (lateral, quasi-lateral or not lateral), and the time interval used to capture each image. Alternating white and grey rows mark stages. Pink rows correspond to events that are present in *D. melanogaster*, but cannot be observed in *C. albipunctata*.
(XLSX)

File S2 **Percentage of cleavage stage embryos from 0 to 100 minutes after egg activation that exhibit a given number of nuclei.** Embryos were collected in 10 min intervals. Data obtained from fixed tissue. *n* indicates the number of embryos assayed at each time interval. The graph in the lower panel is based on the data in the table, and shows the sequential increase in nuclear number over time.
(PDF)

File S3 **Scanning electron micrographs of** ***C. albipunctata*** **blastoderm stage embryos.** A progressively increasing number of nuclei can be seen as cleavage cycles progress from C11 to C14.
(TIF)

Movie S1 **Time-lapse movie covering the entire embryonic development of** ***C. albipunctata*** **.** Time-lapse movie of a *C. albipunctata* embryo taken using a 20× objective and DIC optics under 10S voltalef oil. Lateral view: anterior is to the left, dorsal is up. This movie corresponds to TL2 embryo 1 in Supporting File S1.
(MOV)

Movie S2 **Time-lapse movie covering the blastoderm stage of** ***C. albipunctata*** **.** Time-lapse movie of a *C. albipunctata* embryo taken using a 20× objective and DIC optics under 10S voltalef oil. Lateral view: anterior is to the left, dorsal is up. This movie corresponds to TL2 embryo 2 in Supporting File S1.
(MOV)
