# Supplementary material for: A Staging Scheme for the Development of the Moth Midge Clogmia albipunctata
Source: PLoS One. 2014 Jan 7;9(1):e84422. doi: 10.1371/journal.pone.0084422 (PMC3883683; doi:10.1371/journal.pone.0084422)
Supplement: File S2 — Percentage of cleavage stage embryos from 0 to 100 minutes after egg activation that exhibit a given number of nuclei. Embryos were collected in 10 min intervals. Data obtained from fixed tissue. n indicates the number of embryos assayed at each time interval. The graph in the lower panel is based on the data in the table, and shows the sequential increase in nuclear number over time. (PDF) [file pone.0084422.s002.pdf]

Supporting File S2

time	1 nucleus	2 nuclei	3 nuclei	4 nuclei	5 nuclei	6 nuclei	7 nuclei	8 nuclei	n
10'	98	2	0	0	0	0	0	0	100
20'	89	10	1	0	0	0	0	0	100
30'	12	83	4	1	0	0	0	0	100
40'	2	32	44	21.33	0.67	0	0	0	150
50'	3.33	16	45.33	35.33	0	0	0	0	150
60'	1	11	41	31	14	2	0	0	200
70'	2	5	28.5	47.5	14	2.5	0.5	0	200
80'	0	1	3	50	32	11	3	0	100
90'	0	0.67	10.67	35.33	29.33	22	1.33	0.67	150
100'	3	6	0	6	28	48	5	4	100

Percentage of embryos at every time point that have 1, 2, 3, 4, 5, 6, 7 or 8 nuclei.

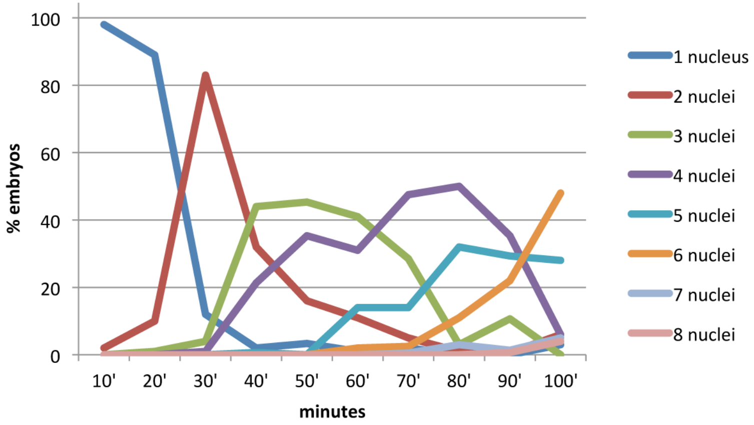

Supporting File S2. Percentage of cleavage stage embryos from 0 to 100 minutes after egg activation that exhibit a given number of nuclei

Supporting File for: Jiménez-Guri, E., Wotton, K. R., Gavilán, B., & Jaeger, J. (2013). A staging scheme for the development of the moth midge *Clogmia albipunctata*. PLoS One.
