# Supplementary figures and images for: A Staging Scheme for the Development of the Moth Midge Clogmia albipunctata
Source: PLoS One. 2014 Jan 7;9(1):e84422. doi: 10.1371/journal.pone.0084422 (PMC3883683; doi:10.1371/journal.pone.0084422)

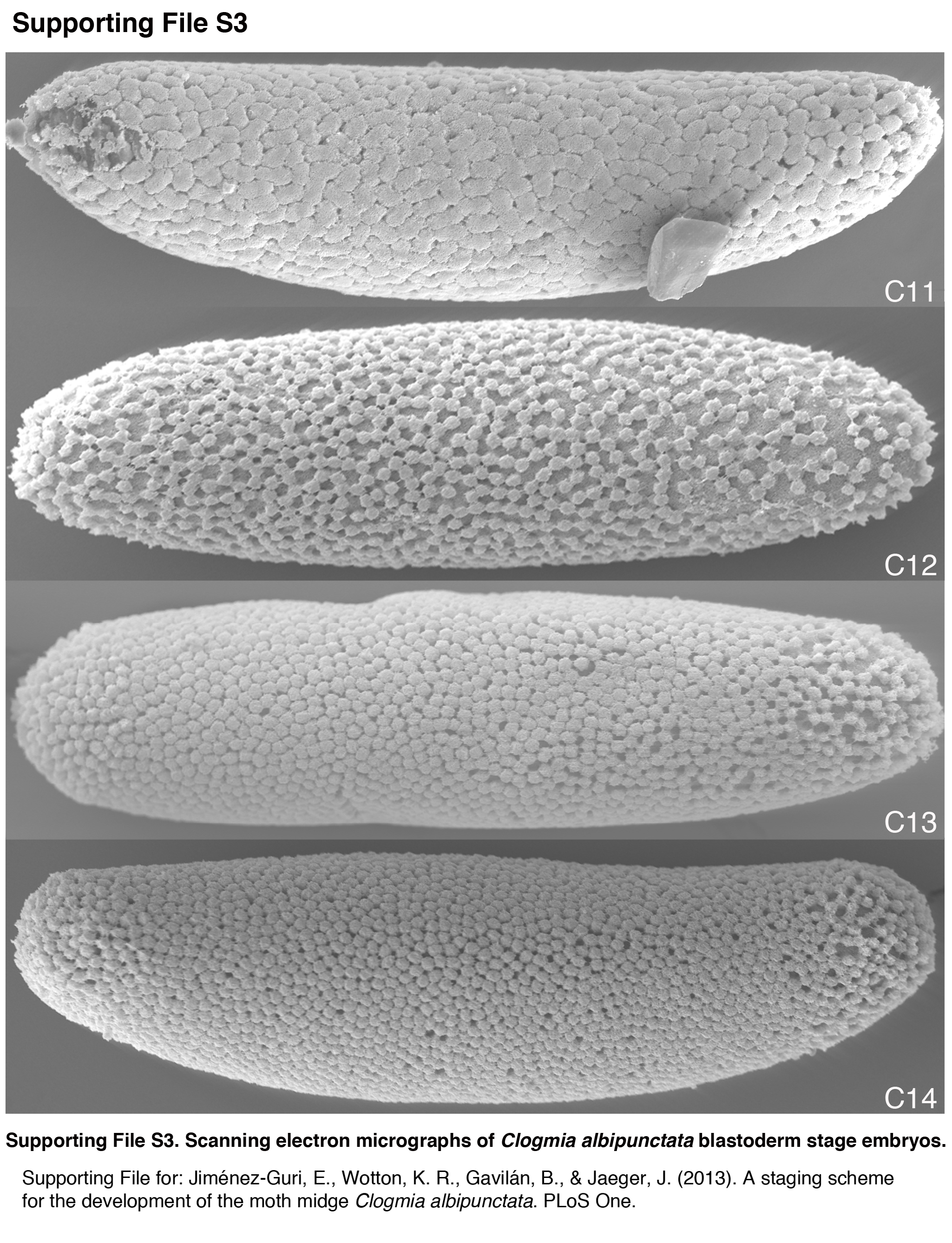

Supplement: File S3 — Scanning electron micrographs of C. albipunctata blastoderm stage embryos. A progressively increasing number of nuclei can be seen as cleavage cycles progress from C11 to C14. (TIF) [file pone.0084422.s003.tif]
